# The Isolation of Scarlet Fever Patients

**Published:** 1897-02-27

**Authors:** 


### The Isolation of Scarlet Fever Patients.

The isolation of cases of infectious disease is becoming so popular that there is some cause for fear lest it should come to be regarded as the beginning and the end of all sanitary endeavour. People are to be found who say, "Let us get every case into hospital, and then we shall be safe." Men of broader views quite understand that the advocacy of so simple a doctrine is but "playing to the gallery." But then does not the gallery contain a very respectable audience, including, indeed, many a medical officer of health? The fact is that isolation hospitals are a useful and in many cases a necessary part of the sanitary service of the country, and the more we arrange our lives in such a manner that no provision is made for the treatment of the sick at home, the more necessary will it become that the sick should go to hospital; but it is not by hospitals that epidemic disease is to be checked. A great and far too common error is to class all infectious diseases together, and to argue from one as to the treatment of the rest; and while we are quite agreed as to the desirability of isolating cases of small-pox, the success of measures taken with that end must not be accepted as in any way supporting the idea that epidemics of other diseases are to be controlled by isolation. A very timely essay on the natural history of scarlet fever, by Dr. John T. Wilson, of Aberdeen, has appeared as a supplement to *Public Health*, in which it is shown how little hospital isolation has had to do with the lowering of the death-rate from that disease, which has been observed during recent years.

The diminution which has taken place in the mortality from scarlet fever since the seventies is enormous, whether we take our statistics from England, from Scotland, or from Ireland. The great epidemics which up to then occurred as ordinary events every five or six years are now things of the past, and if we could believe that the improvement were due to the hospitals which have lately figured so largely in our sanitary system, we should have good reason for satisfaction at their success.

The first shock to such an optimistic conclusion comes from our finding that almost all over the world there has of late been a lessened mortality from scarlet fever, and that whatever its cause this has occurred in places unprovided with isolation hospitals in almost the same proportion as in places where these institutions have been in operation. Hospitals for infectious diseases were for a considerable time possessed only by towns, and by comparatively few even of them in size or number sufficient to be influential. But the decline in the scarlet fever death-rate was not confined to the large towns which had led the way in taking active preventive measures. "Towns without even the nucleus of a sanitary department, and rural districts which made no pretence to sanitary effort of any kind, all participated in the reduction of scarlet fever mortality." In considering the cause, then, of this general reduction, Dr. Wilson says we may almost leave out of account measures for isolation, as the country in general did not enjoy their benefits to any great extent during the period under consideration.

"In Glasgow hospital isolation was largely practised without notification; in Dundee notification was in force, but almost no hospital isolation; in Edinburgh both measures were in operation, while in Leith neither were to any extent in operation. Yet the reduction in scarlet fever mortality was slightly greater in Leith and Dundee than in Edinburgh or Glasgow."

What seems clear is that the factor of importance in the production of a high mortality from scarlet fever is type of disease rather than prevalence, and the facts put forward by Dr. Wilson to show that those conditions of life which lead to a high general mortality lead also to the production of a fatal type of scarlet fever are of much interest.

The assertion has, perhaps, been too readily accepted that scarlet fever is little influenced by general sanitary measures. Dr. Wilson disagrees with this dictum. Arranging the various towns in order according to the fatality of their scarlet fever cases, he shows how accurately this order corresponds with that of their general mortality from all causes. The more extensively unhealthy conditions prevail, as shown by the general death-rate, the more fatal is the type of scarlet fever. All this should teach us not to place too much reliance in the popular doctrine that zymotic disease is to be fought by hospital isolation. These hospitals are of infinite service in helping back to health those who are attacked by the disease. But as a means of lessening the annual loss of life, general measures for the promotion of sanitary and social progress which, while improving general healthiness, also modify the type of scarlet fever, are of infinitely greater service.

